# Abuse and coping strategies among older adults (above 60 years) in Lira district, Northern Uganda: A mixed-methods study protocol

**DOI:** 10.1371/journal.pone.0349559

**Published:** 2026-05-18

**Authors:** Eustes Kigongo, Marvin Musinguzi, Anne Ruth Akello, Freda Amito Oceng, Bosco Opio, Innocent Ojok Opio, Viola Nalwoga, Ritah Bakesiima, Maxson Kenneth Anyolitho, Amir Kabunga, Marc Sam Opollo

**Affiliations:** 1 Department of Health Policy, Planning and Management, Faculty of Public Health, Lira University, Lira, Uganda; 2 Department of Community Health, Faculty of Public Health, Lira University, Lira, Uganda; 3 Department of Environmental Health and Disease Control, Faculty of Public Health, Lira University, Lira, Uganda; 4 Department of Nutrition, Faculty of Public Health, Lira University, Lira, Uganda; 5 Department of Epidemiology and Biostatistics, Faculty of Public Health, Lira University, Lira, Uganda; 6 Department of Psychiatry, Faculty of Medicine, Lira University, Lira, Uganda; Caleb University, NIGERIA

## Abstract

**Background:**

There is a growing number of older adults facing a wide range of abuses by their caregivers and the public. However, there is not only limited information on the outcomes of such abuses but also on the ways that these people employ to cope when faced with such abuses. In this study, we intend to assess the different forms of abuse, estimate their level of abuse, and explore the coping strategies among older adults through a community-based study in a rural setting in northern Uganda.

**Methods:**

The study will employ a community-based, explanatory sequential design. The study will be conducted in Lira district, Lango subregion, Northern Uganda. According to recent (2023) estimates, Lira district has a population of 19,000 older adults (aged over 60) out of a total population of 655,173 people. A sample of 330 participants will be selected by systematic random sampling and interviewed using a researcher-administered questionnaire. Questions on background characteristics, forms of abuse, and copping strategies will be asked. Qualitative data on the experiences of the older adults will be collected through in-depth interview guides to supplement the questionnaire data. The main study outcome is abuse, which will be assessed using the Hwalek-Sengstock Elder Abuse Screening Test (HS-EAST). Data collection will commence in April 2025. The STATA version 17 software will be utilized to perform statistical analysis at univariate, bivariate, and multivariate levels to establish levels of abuse and associated factors. Statistical significance will be set at 5% with a confidence interval of 95%. Qualitative data will be transcribed verbatim and analyzed manually through the thematic method of analysis. The study has obtained approval from the Lira University Research Ethics Committee (LUREC-2023–24).

**Discussion:**

Understanding and recognizing that the elderly suffer harassment, violence, insults, abandonment, isolation, and loneliness is crucial to responding effectively to this phenomenon. Researchers can gain insights into the specific challenges faced by older adults and develop appropriate interventions for prevention and support to improve the wellbeing of older adults, and their families.

## Background

It is now well documented that the older adult population is growing at a rapid rate globally. There will be 1.4 billion and 2.1 billion people who are 60 years of age or older by 2030 and 2050, respectively [1]. The predicted population growth rates in low- and middle-income countries (LMICs) are still higher than those in high-income nations [2]. It is also projected that by 2050, 80% of the world’s older adults will live in LMICs [1], with about 163 million older persons (those over 60 years) in Sub-Saharan Africa alone [3]. However, the growing older adult population faces physical, emotional, and psychological issues [4]. Abuse of older adults, including neglect, is a serious public health issue that affects both developed and developing nations [5]. Elder abuse affects about one in six individuals aged 60 and older worldwide [6].

Older adult abuse, often referred to as elder abuse, mistreatment, or maltreatment, is characterized as a single, repetitive, or improper conduct that takes place in a relationship where there is a trusting expectation and which causes discomfort or harm to an older person [7]. There are different categories of elder abuse, which include the psychological, financial, physical, sexual, and neglect of their welfare by people directly responsible for their care [8]. A review among 44 countries in 2017 reported an average prevalence of elder abuse of 15.7% however, with sparse literature in LMICs [6]. Sub-Saharan Africa reports even higher rates of 64% in South Africa [9], 81% in Kenya [10], and 89% in Uganda [4]. Though staff self-reports indicate a higher prevalence of elder abuse in institutional settings, with up to 64.2% of staff confessing to elder abuse [9], the rates among community settings should not be underestimated [6]. Elder abuse causes injuries, worsens medical problems, and raises hospitalization risk [11]. According to the World Health Organization, those who disclose abuse are twice as likely to die as those who do not [12]. In addition, victims of abuse who had multiple incidents were more likely to be hospitalized, die from any cause, and have poor health than elderly victims who had only one incident [13].

Despite the significant impact of elder abuse on its victims, there is limited knowledge about the coping mechanisms employed in response to such experiences [14]. However, different coping mechanisms, categorized as disengagement and engagement coping, can be employed [15]. The common methods for disengaging include denial, avoidance, and substance abuse. In contrast, engagement coping techniques that aim to lessen or eliminate the stressor or the emotional responses include emotional orientation or problem-focused mechanisms such as asking for emotional support from others [16]. In the aftermath of abuse, victims can easily engage in maladaptive disengagement strategies that further negatively impact their health holistically [17]. A number of studies concerning the lived experience of elder abuse and coping have been conducted in developed countries [14,18]. However, more research is needed, especially in developing countries, to gain a comprehensive picture of how older adults cope with abusive experiences.

Though there is limited information in Uganda, it has been established that a lot of elder abuse occurs in community settings, mostly by family members at home [19]. This has been associated with the age-related instability of social and economic circumstances in Uganda [20]. The study also reported that the current rural displacement, shifting social attitudes, HIV/AIDS, and cultural changes have left the elderly more susceptible to abuse and neglect [21]. Consequently, a recent study in southwestern Uganda showed that, overall, 89.0% of participants had faced some form of abuse [4]. The most prevalent form of elder abuse (86%) was neglect, which was followed by emotional abuse (49%), financial abuse (46.7%), physical abuse (25%), and sexual abuse (6.8%) [4]. Notably, this study focused on prevalence and not coping strategies.

At the national level, Uganda’s Ministry of Gender, Labour and Social Development developed the National Plan of Action for Older Persons (2012–2017) to empower older adults through participation in development programmes and improve their wellbeing. The National Council for Older Persons Act (2013) further established structures for representation and introduced the Social Assistance Grants for Empowerment (SAGE) to provide direct income support. Other initiatives, such as the National Social Protection Policy (2015) and the Special Enterprise Grant for Older Persons (SEGOP), aim to enhance social security and economic inclusion. However, these programmes face challenges, including irregular disbursement, limited coverage, and weak targeting, which undermine their effectiveness in protecting older persons from abuse and neglect [22].

In northern Uganda, older people face a variety of difficulties, such as functional impairment, poor health, unemployment, chronic illness, HIV/AIDS, a lack of social protection institutions, and political unrest [23]. The region also experienced a prolonged civil war that caused a deterioration of most social systems and traditions, including care for the elderly [24]. The region has experienced high rates of domestic violence, particularly in rural areas, affecting many individuals, including older adults, who are exposed to these harmful behaviours [25]. These difficulties lower older people’s quality of life and leave them vulnerable to abuse and neglect from their relatives and peers. There is a dearth of information regarding the well-being of older adults in Northern Uganda, particularly relating to abuse, neglect, and coping strategies. While previous research from Western Uganda highlights a higher prevalence of abuse including neglect, sexual, emotional, physical and financial abuse [26], similar studies are scarce in Northern Uganda, necessitating the current study to address this gap.

### Research questions

1. What is the prevalence of abuse among older adults in Lira district, Northern Uganda?
2. What are the different forms of abuse among older adults in Lira district, Northern Uganda?
3. What factors are associated with abuse among older adults in Lira district, Northern Uganda?
4. What coping strategies do older adults in Lira district employ to deal with abusive experiences?

### Study objectives

1. To determine the prevalence of abuse among older adults in Lira district, Northern Uganda.
2. To identify the different forms of abuse among older adults in Lira district, Northern Uganda.
3. To establish factors associated with abuse among older adults in Lira district, Northern Uganda.
4. To explore coping strategies employed by older adults in Lira district in dealing with abusive experiences.

### Study significance

The findings of the study have implications for social work research, education, practice, and policy. The study will contribute to the body of knowledge on vulnerabilities that predispose older adults to abuse in the Ugandan context. The data will be available for use by government and non-government organizations, community and faith-based organizations that are engaged in ensuring wellbeing of older adults. Additionally, the findings will guide authorities in adjusting policies on elderly care and support. During data collection, all participants identified with mental health challenges requiring counselling or mental health services will be referred to appropriate care. Finally, this study will result in reporting findings that will provide evidence for advocacy campaigns to prevent abuse among old people.

## Materials and methods

### Study design

The study will employ an explanatory sequential design to collect and analyze data from older adults in the selected communities. Firstly, quantitative data on the prevalence and forms of abuse, and associated factors will be collected, followed by qualitative data on the experiences of the older adults and their coping mechanisms while dealing with abuse. The design will guide the understanding of the prevalence, different forms of abuse and explore the different reasons for abuse as well as coping strategies employed.

### Study area and setting

The study will be conducted in Lira district, Lango subregion, Northern Uganda. Lira district is bordered by Pader district to the north, Otuke district to the northeast, Alebtong district to the east, Dokolo district to the southeast, Apac district to the southwest, and Kole district to the west. The district is located approximately 337 kilometers by road, north of Kampala, Uganda’s capital city. The district is among the districts that suffered the close to two-decade insurgency that caused displacement and disruption of social norms and practices, a challenge that contributes to elder abuse [24]. Due to abandonment by their families, many old people in Lira district have resorted to begging to sustain their basic needs [27].

### Study population

The study population are the older adults in Lira district, Northern Uganda. According to the Ministry of Gender, Labor, and Social Development, an older adult is a person aged above 60 years. The accessible population are the older adults that will be found at home in the selected sub counties at the time of data collection. Data in 2023 indicates that Lira district has an estimated population of 19,000 older adults aged over 60 years of a total population of 655,173 people, from 15,589 old adults of 408,043 people in 2014 [28]. However, recent estimates in 2023 indicate that the population of older adults in Lira district approximates 19,008.

### Inclusion criteria

We will include all older adults aged 60 years and older living within Lira District. Additionally, only participants who have been residents for the past six months, and present in the household at the time of data collection will be considered eligible for participation. Eligible participants should consent to participate in the study.

### Exclusion criteria

Those who are not willing to provide the required data will be excluded. Also, those who will be critically ill, too weak to be interviewed, or who cannot talk on their own will be excluded. Respondents who are severely cognitively impaired or have incoherent communication will be excluded. This will help to minimize recall bias that might arise due to common dementia among older people. In case there is more than one eligible participant in a household, only one will be selected randomly.

### Sample size determination

The study has employed the Kish Leslie (1965) formula to calculate the sample size [29]. Using the 89% prevalence of elder abuse in a recent study in western Uganda [26], a standard Z score of 1.96, corresponding to 95% level of confidence, and a significance level of 5% have been employed to compute the minimum sample size. Additionally, due to the use of multi-stage sampling techniques in obtaining participants, a design effect factor of 2.0 has also been factored in the calculation [30]. This produces a final sample size of 330 participants after increasing by 10% to account for non-response. For qualitative data, we expect to conduct in-depth interviews with 20–30 participants, however, the final sample will be achieved through data saturation [31]. Data saturation will be reached when no new concepts, ideas or insights are yielding with each net respondent repeating what has been said.

### Sampling methods and participant recruitment

The study will use a multi-stage sampling procedure to recruit and enrol respondents. The district comprises seven sub-counties, out of which three will be selected through simple random sampling. This will involve writing the names of all sub-counties on small pieces of paper, folding them, mixing them thoroughly in a container, and randomly picking three with replacement. From each selected sub-county, two parishes will be chosen, followed by two villages/cells from each parish, also using the simple random sampling method described above. This process will yield a total of 12 villages. Village Health Teams (VHTs) will assist in accessing older adults in each village by enumerating all eligible households with individuals aged 60 years and above, which will serve as the sampling frame for each village. The number of participants to be selected from each village will be proportional to the number of eligible individuals or households in that village. A systematic random sampling procedure will then be applied to identify participants. After obtaining the eligible participants list (N), a skip interval (K) will be determined by dividing the sample frame by the sample size (n). The first participant will be selected through simple random sampling from the first K positions on the eligible participants list. Subsequent participants will be selected as the Kth position on the eligible participants list. This will be performed until the required sample (n) will be obtained.

Participants for qualitative interviews will be selected by purposive sampling. Within each household, the eligible participant will be approached by the research assistant with written consent. Only those who consent will be interviewed. Through the quantitative interviews, prospective participants for the in-depth interviews will be identified to have a mix of respondents with severe and minimal abuse to allow for adequate exploration of contributing factors.

### Data collection tools and methods

Different data collection tools and methods will be employed for both quantitative and qualitative data. For quantitative data, an interviewer-administered questionnaire will be used through face-to-face interviews. The questionnaire will comprise of different sections including the demographic factors, questions on abuse and different forms of abuse, community and health system factors associated with abuse, and coping strategies. The Hwalek-Sengstock Elder Abuse Screening Test (HS-EAST) scale will be used to collect data on elder abuse. This tool comprises of 15-items intend to detect circumstances considered as correlates of elder abuse, and estimate the prevalence of abuse [32]. Data on coping strategies will be collected using the Brief COPE tool [33]. The Brief COPE tool consists of 28 items on a 4-point positive Likert scale. These items are recorded to generate three categories: problem-focused coping, emotion-focused coping, and avoidant copying. The different forms of abuse will be assessed by a tool originally developed by the National Research Council (US) Panel on Elder Abuse and Neglect [34]. All the tools we intend to use in this start have good psychometric properties and have been validated for use in different settings, including some related studies in Uganda [26,35]. The tool will be pretested in a non-participant population of older adults before actual data collection and revised iteratively. To collect qualitative data, an in-depth interview guide will be employed. This guide will be developed using insights from quantitative results and pretested for use. To enhance the data collection quality, audio-recording will be done complemented by extended filed notes taking when the participants permit. We anticipate data collection to commence in April 2026.

### Data collection procedures

All the data will be collected physically from the older adults at designated private places at their homes. Face-to-face interviews will be conducted by trained research assistants. This will ensure the collection of quality data but also prevent the unnecessary missing of key data. The research assistants will be introduced to the homes of the older adults, or where they are cared for by a Village Health Team (VHT) member who represents the respective villages. During interviews, all third parties will not be allowed at the designated private place unless permitted by the older adult under interview. If more than one older person is found in a home, one will be selected by simple random sampling. The questionnaires will also be anonymized, and all the data collected will be kept with the utmost confidentiality through lock and key for hard copies at the Faculty of Public Health and passwords for softcopy data. To ensure comprehension and ease of interviews, all the tools will be administered in the local dialect (Luo) and are expected to take about half an hour. Based on the preliminary findings of the quantitative data, we shall revise the pre-designed interview guide to conduct qualitative interviews. The qualitative interviews will be audio recorded with consent, complemented by extensive field notes. After the interviews, the older adults will be compensated for their time with a bar of soap and a kilogram of sugar each.

### Study outcomes and measurement

The main outcome of the study is abuse of older adults and will be measured as a binary outcome of yes or no, following the guidance of the HS-EAST tool [36]. The secondary outcomes of the study include physical, sexual, emotional, and financial abuse, as well as neglect. These will be measured as binary outcomes.

The independent variables are categorized into three categories: individual factors, community factors, and support system-related factors. The individual factors comprise demographic and other background characteristics including age, sex, education level, marital status, religion, income status, household size, and characteristics of the caregivers. The community factors comprise community norms and attitudes, social networks, and community awareness. The support system factors comprise of caregiver training and support, access to health services, and legal protection and reporting mechanisms. The Brief COPE tool, a 28-item checklist, will be used to collect data on coping strategies. These will be summarized as problem-focused coping, emotion-focused coping and avoidant copying.

For the qualitative part of the study, we shall explore the experiences and perspectives of older adults who have faced severe abuse. Additionally, the different ways that they employ to cope with abusive experiences will be explored.

### Data management

Data will be collected by five trained research assistants who are trained in social work to ensure the quality of the data collection. On-field, after collection and after entry of data, editing will be conducted. During field collection, research assistants will check for completeness of questionnaires before leaving the respondents’ home. Every day after filed collection, the research assistants will have a meeting with the principal investigator to discuss any issues or challenges encountered during the day, and cross check the questionnaires for errors that could have been made. All the hard copies will be handed over to the principal investigator, who will keep them under lock and key at the Faculty of Public Health. The quantitative data will be entered into Microsoft Excel (2013) by two data clerks. The data will be checked for missing values by identifying the type of missingness. Missing data will be handled by either deleting some records where one or more of the key variables are missing, or by computing the mean or median of the nearby point. The validated data will be exported to STATA version 17 for formal analysis. Qualitative data will be audio-recorded during interviews and transcribed verbatim. During interviews, extensive field notes will be made to ensure that all the non-verbal cues are captured. Research assistants will be tasked with translation and transcription of the data. For quality, a person fluent in the Luo dialect will be recruited to do similar translation and transcription and comparisons will be made. All the data will be destroyed after 5 years.

### Data analysis

Quantitative and qualitative data analysis approaches will be employed appropriate for the study objectives. The first two objectives for prevalence of abuse, and identification of the different forms of abuse will be analyzed using descriptive analysis. These results will be summarized as frequencies and percentages and presented using appropriate charts and tables.

For objective three of associated factors, bivariate and multivariate analysis will be performed. A Pearson chi-square (or Fisher’s exact) test will be performed to assess the association between dependent and independent variables. Variables with p < 0.2 at bivariate, and other plausible variables from the literature will be moved for multivariate analysis. This will be performed after a careful assessment of the underlying assumptions. Independent variables with p < 0.05 will be deemed statistically significant with the dependent variable. Exploratory factor analysis will be employed to understand the patterns and underlying structures among the variables measuring coping strategies, and to reduce large numbers of observed variables into a smaller set of latent factors [37]. Subsequently, principal components analysis and logistic regression will be conducted.

For the final objective on coping strategies, qualitative analysis will be performed manually using Braun and Clarke’s six-step approach to thematic analysis. First, trained research assistants will transcribe the interviews verbatim, and the transcripts will be reviewed for accuracy by the principal investigator. The coding and analysis will then be conducted by a separate team of researchers to minimize bias. The researchers will read the transcripts thoroughly to become immersed in the data. To identify key concepts and patterns, a hybrid approach, combining deductive coding based on a priori themes informed by the study objectives and relevant theoretical concepts, with inductive coding to capture new and emerging themes from the data. Overarching themes will be developed through iterative discussions and refined collaboratively to ensure they accurately reflect participants’ perspectives. To reduce intercoder bias, at least two coders will independently code a subset of transcripts, compare results, and resolve discrepancies through consensus meetings. The final themes will be validated against coded extracts and discussed in team workshops and participant validation sessions to enhance credibility and inclusivity.

Quantitative and qualitative results will be analyzed and presented separately, with integration occurring in the discussion and interpretation of results section.

### Ethical considerations

The study obtained ethical approval from the Lira University Research Ethics Committee (LUREC-2023–24). Before recruiting participants, informed consent will be obtained through signing or a thumb print for those who cannot write. The research assistants will carefully explain the study to eligible participants, including its objectives, procedures, timeframe, benefits, risks, and participants’ rights using the language that the prospective participant understands. It will clearly be explained that the participants can opt out any time and no maltreatment will be given to anyone who decides not to participate. Data collection will prioritize privacy by conducting interviews in participants’ homes or other private spaces and ensuring confidentiality through the anonymization of questionnaires, secure storage under lock and key, and password-protected data. All respondents will be treated with respect, and as a compensatory benefit, each participant will receive a bar of soap and a kilogram of sugar after the interview. In case respondents report serious forms of abuse, there will be psychologists on the teams that will help with provision of psychotherapy, and additionally, the cases will be referred for appropriate management.

### Study limitations

Some of the questions require a self-reported response, which can potentially introduce response bias. Additionally, some of the respondents may not feel free to discuss their issues in fear of prospective mistreatment. However, the research assistants will assure confidentiality, and both male and female research assistants of middle age will be employed to conduct interviews.

## Discussion

Understanding and recognizing that the elderly suffer harassment, violence, insults, abandonment, isolation, and loneliness is crucial to responding effectively to this phenomenon. Researchers can gain insights into the specific challenges faced by older adults and develop appropriate interventions for prevention and support to improve the wellbeing of older adults, and their families.

## Supporting information

S1 Data Data collection tool. (DOCX)
