# Supplementary material for: Abuse and coping strategies among older adults (above 60 years) in Lira district, Northern Uganda: A mixed-methods study protocol
Source: PLoS One. 2026 May 18;21(5):e0349559. doi: 10.1371/journal.pone.0349559 (PMC13183227; doi:10.1371/journal.pone.0349559)
Supplement: S1 Data — (DOCX) [file pone.0349559.s001.docx]

**Data collection tool**

| **A** | **DEMOGRAPHIC CHARACTERISTICS** | **RESPONSES** | | | |
| --- | --- | --- | --- | --- | --- |
| A1 | What is your age in completed years? |  | | | |
| A2 | What is your sex? | 1. Male 2. Female | | | |
| A3 | What is your marital status? | 1. Single 2. Married 3. Separated 4. Widowed | | | |
| A4 | What is your religious affiliation? | 1. Catholic 2. Anglican 3. Muslim 4. Pentecostal 5. Other | | | |
| A5 | Do you have any formal education? | 1. Yes 2. No | | | |
| A6 | If Yes in A5 above, what is your highest level of education? | 1. Primary 2. Secondary 3. Tertiary | | | |
| A7 | In which division do you live? | 1. Erute North 2. Erute South | | | |
| A8 | Whom do you stay with? | 1. Alone 2. Partners 3. Other (specify) | | | |
| A9 | Do you have any source of income? | 1. Yes 2. No | | | |
| A10 | If Yes IN A9 above, what is your main source of income? | 1. Business 2. Formal support 3. Begging 4. Other (specify) | | | |
| **B** | **ABUSE (HS-EAST 15-items)** |  | | | |
| B1 | Do you have anyone who spends time with you, taking you shopping or to the doctor? | 1. Yes 2. No | | | |
| B2 | Are you helping to support someone? | 1. Yes 2. No | | | |
| B3 | Are you sad or lonely often? | 1. Yes 2. No | | | |
| B4 | Who makes decisions about your life—like how you should live or where you should live? | 1. Myself 2. Someone else | | | |
| B5 | Do you feel uncomfortable with anyone in your family? | 1. Yes 2. No | | | |
| B6 | Can you take your own medication and get around by yourself? | 1. Yes 2. No | | | |
| B7 | Do you feel that nobody wants you around? | 1. Yes 2. No | | | |
| B8 | Does anyone in your family drink a lot? | 1. Yes 2. No | | | |
| B9 | Does someone in your family make you stay in bed or tell you you’re sick when you know you’re not? | 1. Yes 2. No | | | |
| B10 | Has anyone forced you to do things you didn’t want to do? | 1. Yes 2. No | | | |
| B11 | Has anyone taken things that belong to you without your O.K.? | 1. Yes 2. No | | | |
| B12 | Do you trust most of the people in your family? | 1. Yes 2. No | | | |
| B13 | Does anyone tell you that you give them too much trouble? | 1. Yes 2. No | | | |
| B14 | Do you have enough privacy at home? | 1. Yes 2. No | | | |
| B15 | Has anyone close to you tried to hurt you or harm you recently? | 1. Yes 2. No | | | |
| **C** | **FORMS OF ABUSE** |  | | | |
| E1 | Has anyone ever verbally attacked, scolded, or yelled at you so that you felt afraid for your safety, threatened or intimidated? | 1. Yes 2. No | | | |
| E2 | Has anyone ever made you feel humiliated or embarrassed by calling you names such as stupid, or telling you that you or your opinion was worthless? | 1. Yes 2. No | | | |
| E3 | Has anyone ever forcefully or repeatedly asked you to do some-thing so much that you felt harassed or coerced into doing something against your will? | 1. Yes 2. No | | | |
| E4 | Has anyone close to you ever completely refused to talk to you or ignored you for days at a time, even when you wanted to talk to them? | 1. Yes 2. No | | | |
| P1 | Has anyone ever hit you with their hand or object, slapped you, or threatened you with a weapon? | 1. Yes 2. No | | | |
| P2 | Has anyone ever tried to restrain you by holding you down, tying you up, or locking you in your room or house? | 1. Yes 2. No | | | |
| P3 | Has anyone ever physically hurt you so that you suffered some degree of injury, including cuts, bruises, or other marks? | 1. Yes 2. No | | | |
| S1 | Regardless of how long ago it happened or who made the advances, has anyone ever made you have sex or oral sex by using force or threatening to harm you or someone close to you?” | 1. Yes 2. No | | | |
| S2 | For females - Has anyone ever touched your breasts or pubic area or made you touch his penis by using force or threat of force? | 1. Yes 2. No | | | |
| S3 | For males - Has anyone ever touched your pubic area or made you touch their pubic area by using force or threat of force? | 1. Yes 2. No | | | |
| S4 | For females - Has anyone ever forced you to undress or expose your breasts or pubic area when you didn’t want to? | 1. Yes 2. No | | | |
| S5 | For males - Has anyone ever forced you to undress or expose your pubic area when you didn’t want to? | 1. Yes 2. No | | | |
| N1 | Do you need someone to help you get to the places you need to go, for example, do you need someone to drive you to the grocery store, a place of worship, the doctor? | 1. Yes 2. No | | | |
| N2 | Do you need someone to make sure you have enough food, medicines, or any other things you need in your house? | 1. Yes 2. No | | | |
| N3 | Do you need someone to help you with household things, like cooking meals, helping you eat, or making sure you take the correct medicines each day? | 1. Yes 2. No | | | |
| N4 | Do you need someone to help you with house cleaning or yard work? | 1. Yes 2. No | | | |
| N5 | Do you need someone to help you get out of bed, get showered, or get dressed? | 1. Yes 2. No | | | |
| N6 | Do you need someone to make sure your bills get paid? | 1. Yes 2. No | | | |
| F1 | Is there someone who helps you take care of your finances, or is there someone other than yourself who makes decisions about your money and your property, either with or with-out your approval? | 1. Yes 2. No | | | |
| F2 | Does that person ask for your PERMISSION before deciding to spend your money or sell your property? | 1. Yes 2. No | | | |
| F3 | Do you feel like that person makes good decisions about your finances? | 1. Yes 2. No | | | |
| F4 | “Do you have the copies of paperwork for the financial decisions they make or can you get copies if you wanted them? | 1. Yes 2. No | | | |
| F5 | Has that person ever forged your signature without your permission to sell your property or to get money from your accounts? | 1. Yes 2. No | | | |
| F6 | Has that person ever forced or tricked you into signing a document so that they would be able to get some of your money or possessions? | 1. Yes 2. No | | | |
| **D** | **COMMUNITY AND SUPPORT SYSTEM** |  | | | |
| D1 | Do you entirely depend on another person for daily living? | 1. Yes 2. No | | | |
| D2 | How is your relationship with your caregiver? | 1. Good 2. Poor | | | |
| D3 | How old is your main caretaker or caregiver? |  | | | |
| D4 | What is the sex of your caretaker or caregiver? | 1. Male 2. Female | | | |
| D5 | Do you usually take any drugs such as alcohol excessively? | 1. Yes 2. No | | | |
| D6 | Do you have friends that you spend time with daily? | 1. Yes 2. No | | | |
| D7 | How is your relationship with your family members? | 1. Good 2. Poor | | | |
| D8 | Do you have easy access to services like police and courts of law? | 1. Yes 2. No | | | |
| D9 | Do you have access to healthcare services? | 1. Yes 2. No | | | |
| E | **COPING STRATEIES** (1=I have not been doing this at all; 2=A little bit; 3=A medium amount; 4=I have been doing this a lot) | 1 | 2 | 3 | 4 |
| E1 | I've been turning to work or other activities to take my mind off things |  |  |  |  |
| E2 | I've been concentrating my efforts on doing something about the situation I'm in |  |  |  |  |
| E3 | I've been saying to myself "this isn't real" |  |  |  |  |
| E4 | I've been using alcohol or other drugs to make myself feel better |  |  |  |  |
| E5 | I've been getting emotional support from others |  |  |  |  |
| E6 | I've been giving up trying to deal with it |  |  |  |  |
| E7 | I've been taking action to try to make the situation better |  |  |  |  |
| E8 | I've been refusing to believe that it has happened |  |  |  |  |
| E9 | I've been saying things to let my unpleasant feelings escape |  |  |  |  |
| E10 | I’ve been getting help and advice from other people |  |  |  |  |
| E11 | I've been using alcohol or other drugs to help me get through it |  |  |  |  |
| E12 | I've been trying to see it in a different light, to make it seem more positive |  |  |  |  |
| E13 | I’ve been criticizing myself |  |  |  |  |
| E14 | I've been trying to come up with a strategy about what to do |  |  |  |  |
| E15 | I've been getting comfort and understanding from someone |  |  |  |  |
| E16 | I've been giving up the attempt to cope |  |  |  |  |
| E17 | I've been looking for something good in what is happening. |  |  |  |  |
| E18 | I've been making jokes about it |  |  |  |  |
| E19 | I've been doing something to think about it less, such as going to movies, watching TV, reading, daydreaming, sleeping, or shopping |  |  |  |  |
| E20 | I've been accepting the reality of the fact that it has happened |  |  |  |  |
| E21 | I've been expressing my negative feelings |  |  |  |  |
| E22 | I've been trying to find comfort in my religion or spiritual beliefs |  |  |  |  |
| E23 | I’ve been trying to get advice or help from other people about what to do |  |  |  |  |
| E24 | I've been learning to live with it |  |  |  |  |
| E25 | I've been thinking hard about what steps to take |  |  |  |  |
| E26 | I’ve been blaming myself for things that happened |  |  |  |  |
| E27 | I've been praying or meditating |  |  |  |  |
| E28 | I've been making fun of the situation |  |  |  |  |
